# The Impact of Hyperbaric Oxygen Therapy on Functional and Structural Plasticity in Rats With Spinal Cord Injury

**DOI:** 10.1002/brb3.70196

**Published:** 2024-12-11

**Authors:** Xinyi Yang, Zhongyue Wu, Huimin Lai, Lingling Chen, Dairong Cao, Fang Liu

**Affiliations:** ^1^ Department of Radiology The First Affiliated Hospital of Fujian Medical University Fuzhou Fujian China; ^2^ Department of Hyperbaric Oxygen The First Affiliated Hospital of Fujian Medical University Fuzhou Fujian China; ^3^ Fujian Key Laboratory of Precision Medicine for Cancer The First Affiliated Hospital Fujian Medical University Fuzhou Fujian China; ^4^ Department of Radiology The First Affiliated Hospital of Fujian Medical University Binhai Campus Fuzhou Fujian China; ^5^ Department of Hyperbaric Oxygen The First Affiliated Hospital of Fujian Medical University Binhai Campus Fuzhou Fujian China

**Keywords:** hyperbaric oxygen treatment, research resource identifiers, resting‐state functional magnetic resonance imaging, spinal cord injury

## Abstract

**Introduction:**

Spinal cord injury (SCI) can result in sensory and locomotor function loss below the injured segment. Hyperbaric oxygen therapy (HBOT) has been proven to alleviate SCI. This study aims to establish a reproducible rat model of SCI and investigate the impact of HBOT on alterations in brain neuronal activity and neuromotor function in this experimental rat SCI model using resting‐state functional magnetic resonance imaging (rs‐fMRI).

**Methods:**

This is a prospective randomized controlled animal trial. A total number of 27 female SD rats were randomly divided into three groups: sham (*n* = 9), SCI (*n* = 9), and HBO (*n* = 9). rs‐fMRI was utilized to assess regional homogeneity (ReHo) values and functional connectivity (FC) strength over the whole brain with the motor cortex as seeds. Correlation between neuroimaging characteristics and behavioral assessment was calculated. We examined Nissl body, NeuN, and caspase‐3 expression in relevant brain regions.

**Results:**

Following SCI, reduced ReHo values were observed in the left primary somatosensory cortex, left striatum, right agranular insular cortex, and partial cortex in the limbic system, which was reversed after HBOT. HBOT could increase FC strength between the motor cortex and other brain regions, including the left secondary motor cortex, right basal forebrain region, bilateral primary somatosensory cortex, bilateral thalamus, and another partial cortex in the limbic system. BBB scale scores showed that HBOT promoted motor function recovery in SCI rats. The ReHo and FC values in all positive clusters were positively correlated with BBB scores. By histopathological analysis, our study found that HBOT could reduce apoptotic proteins, increase the number of neurons, and protect neuronal function in brain regions with significant ReHo and FC alteration in SCI rats.

**Conclusion:**

This study reveals that HBOT facilitates functional and structural plasticity in the brain, contributing to the recovery of motor function in rats with SCI.

## Graphical abstract

After hyperbaric oxygen (HBO) therapy, improved motor function, increased cerebral cortical activity, and enhanced functional connection strength are observed in spinal cord injury (SCI) rats. In addition, HBO therapy could reduce apoptotic proteins, increase the number of neurons, and protect neuronal function in brain regions with significant alterations in regional homogeneity and functional connectivity in SCI rats.

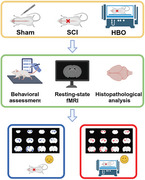

## 1 Introduction

Spinal cord injury (SCI) represents a grave complication of spinal injuries, severely affecting sensory and locomotor functions below the injured site (H. Suzuki and Sakai 2021). SCI can be generally categorized into primary and secondary injuries (Rouanet et al. 2017). Primary injury, irreversible in nature, results from immediate mechanical trauma to the spinal cord due to external forces, whereas secondary injury encompasses subsequent pathophysiological processes, such as apoptosis, oxidative stress, ischemia, edema, inflammation, and excitotoxicity, occurring after the initial mechanical trauma (Kwon et al. 2004; David et al. 2019). Therefore, the primary goal of clinical SCI treatment is to minimize secondary damage and promote the repair and regeneration of the injured spinal cord.

Currently, no specific therapies exist for secondary injury. The prevailing approach to addressing secondary injury involves early surgical intervention combined with neuroprotective drugs (e.g., methylprednisolone and gangliosides) and electrical stimulation (Huang et al. 2020). However, while surgical treatment can alleviate spinal cord compression, its effectiveness in preventing the progression of secondary injuries is limited (Huang et al. 2020). Neuroprotective drugs are no longer recommended in current guidelines due to their low efficacy and high complications (Hurlbert et al. 2015). In addition, electrical stimulation is highly traumatic, dangerous, and painful. Therefore, there exists an urgent clinical imperative for an adjunctive treatment method that is less harmful to the human body and more effective to compensate for surgical treatment's shortcomings.

Hyperbaric oxygen (HBO) therapy presents a non‐invasive treatment modality. In the HBO treatment of SCI, 2.0 absolute atmosphere (ATA) is a commonly chosen treatment pressure (Siglioccolo et al. 2024; Z. Zhang et al. 2022; Z. C. Sun et al. 2022; Feng and Li 2017). At 2.0 ATA, the plasma oxygen partial pressure rises above 1110 mmHg to increase tissue and cellular oxygenation (Efrati et al. 2013). Moreover, HBO treatment reduces secondary SCI damage through mechanisms such as increasing the diffusion distance of oxygen, inhibiting lipid peroxidation, mitigating inflammatory responses, and decreasing cell apoptosis (L. Sun et al. 2019; H. Chen et al. 2021; Ying et al. 2019). Earlier findings have also indicated that HBO treatment can enhance motor function recovery after SCI (Y. Zhao et al. 2020). B. Zhao et al. (2017) demonstrated that HBO treatment improves learning and memory abilities by reducing neuronal apoptosis in the brain and promoting dendritic reconstruction. In a study involving chronic stroke patients, HBO treatment was found to promote motor function recovery. Furthermore, resting‐state functional magnetic resonance imaging (fMRI) showed increased activation of the bilateral supplementary motor cortex and premotor cortex (Catalogna et al. 2023).

Neuroplasticity is fundamental to SCI treatment (Wallwork et al. 2016; Sigmundsson, Dybendal, and Grassini 2022). Resting‐state fMRI can monitor spontaneous neuronal activity under conditions closely resembling physiological states, facilitating quantitative analysis of functional connections among neurons within and across different cerebral cortices. Previous research has shown that SCI leads to not only direct damage to the spinal cord but also atrophy or apoptosis of cortical neurons at the cerebral level (Hains, Black, and Waxman 2003; Nielson, Strong, and Steward 2011). Zhu et al. (2015) demonstrated a reduction in regional homogeneity (ReHo) values in the bilateral primary motor cortex and primary somatosensory cortex following acute SCI. Hou et al. (2014) reported a decrease in amplitude of low‐frequency fluctuations (ALFF) values in the bilateral primary sensorimotor cortex while observing an increase in ALFF values in the bilateral cerebellum and right preorbital cortex in patients with SCI compared with healthy controls.

However, most previous research has been limited to clinical studies, and some studies have reported contradictory results (Karunakaran et al. 2020; Nakanishi et al. 2021; Zheng et al. 2021; Kim et al. 2022). These discrepancies among studies may stem from variations in injury severity, affected spinal levels, duration since injury, and treatment modalities employed in clinical settings. Therefore, this study aims to establish a reproducible rat model of SCI and investigate the impact of HBO treatment on alterations in brain neuronal activity and neuromotor function in this experimental rat SCI model using resting‐state fMRI.

## 2 Methods

### 2.1 Animals

Female Sprague‐Dawley (SD) rats weighing 220–240 g were obtained from the Animal Experiment Centre of the Fujian Medical University (Fuzhou, Fujian, China). The animals were kept in hygienic cages with controlled temperatures between 22°C and 25°C, following a 12‐h light/dark cycle, and provided with sufficient food and water. Approval for the animal protocol was granted by the Ethics Committee of the Fujian Medical University (IACUC FJMU 2022‐0879). All procedures were conducted to minimize animal usage and reduce animal suffering.

### 2.2 Spinal Cord Injury Model

Referring to our previous study, female SD rats were used to create the SCI models (F. Liu et al. 2019). Rats were anesthetized by intraperitoneal injection of 2% sodium pentobarbital (50 mg/kg). Following disinfection, the skin, subcutaneous tissue, and paravertebral muscles were separated to perform laminectomy on the T9–11 vertebra to expose the spinal cord. SCI was induced at the T10 level using the New York University impactor (Model III, W.M.Keck/USA) by releasing a 10 g rod from 50 mm height. The bladder was manually compressed twice a day until the ability to urinate returned. Rats in the sham group received laminectomy only.

### 2.3 Study Design

A total number of 27 rats were randomly allocated to either the sham group (received laminectomy only, *n* = 9), the SCI group (received SCI, *n* = 9), or the HBO group (received SCI and HBO treatment, *n* = 9). Behavioral assessment and resting‐state fMRI were performed on the seventh day after the model was established.

### 2.4 HBO Treatment

Rats in the HBO group were treated with 60 min of HBO therapy (PO_2_ = 95%–100% at 2.0 ATA) daily in an animal HBO chamber (DC0325J‐X, Yantai Moon Group Co. Ltd., Shandong, China) after injury until the time point. The pressure in the chamber was slowly raised to 2.0 ATA for 10 min, after 60 min of HBO therapy, then lowered to normal air pressure (PO_2_ = 21% at 1.0 ATA) for another 10 min. The chamber was kept at 22–25°C and a 62%–68% humidity level. Rats in the sham and SCI groups were exposed to normobaric air conditions.

### 2.5 Behavioral Assessment

The Basso, Beattie, and Bresnahan (BBB) locomotor scale was conducted in an open field, focusing on the movement of the hind limbs, particularly gait and coordination. Each group was evaluated on the seventh day post‐surgery through double‐blind, independent observations, with the results recorded as previously outlined (F. Liu et al. 2019). Higher scores on the BBB scale indicate better health and behavior in rats.

### 2.6 MRI Data Acquisition

Referring to previous literature, all experiments were conducted in a 70/20 Bruker Biospec MRI scanner (7.0T; Bruker Biospin, Germany) (Dai et al. 2023). Rats were anesthetized with isoflurane and positioned in a prone position on a bed with elastic tape securing them. Anesthetic gas was administered continuously through a nasal cannula, and body temperature was maintained at 37°C.

The acquisition parameters were as follows: T2‐weighted images (T2WI) were acquired using the rapid acquisition with relaxation enhancement sequence (repetition time [TR] = 4200 ms, effective echo time [TE] = 35 ms, field of view [FOV] = 32 × 32 mm, image size = 256 × 256, slices = 32, slice thickness = 1 mm). The resting‐state fMRI signals were acquired using an echo‐planar imaging (EPI) sequence (TR = 2000 ms, TE = 14 ms, FOV = 32 × 32 mm, repetitions = 300, image size = 64 × 64, slices = 32, slice thickness = 1 mm).

### 2.7 Functional Image Processing

Resting‐state fMRI data were preprocessed and statistically analyzed using SPM12 software (https://www.fil.ion.ucl.ac.uk/spm/software/spm12, RRID: SCR_007037) in MATLAB (version 2022b, RRID: SCR_001622). The first 20 volumes were removed, and images were corrected for slice timing and head motion. Data were then standardized to the SIGMA in vivo rat brain template. Eight nuisance covariates, including the white‐matter signal, the cerebrospinal fluid signal, and six head motion parameters, were regressed to eliminate the possible variances in the time course of each voxel.

### 2.8 ReHo Calculation

ReHo assesses local temporal similarity between a voxel and its neighbors to measure functional synchronization within nearby brain regions. The ReHo value for the central voxel was determined by Kendall's coefficient of concordance (KCC). To create individual ReHo maps, KCC values were calculated for each voxel and its nearest neighbors, then smoothed with Gaussian kernel by 0.6 mm full width of half maximum. Statistical analyses were conducted at the cluster level and utilized one‐way analysis of variance (ANOVA) to identify variations among groups. Only clusters that surpassed a cluster‐level threshold corrected at a false discovery rate (FDR) method of *p* < 0.05 were plotted.

### 2.9 Functional Connectivity Analysis

Using the motor cortex (comprising primary and secondary motor cortex) as a seed point, voxel‐wised functional connectivity (FC) analysis was performed to assess alterations in whole‐brain FC of brain regions. Subsequently, the resulting values underwent Fisher's *r*‐to‐*z* transformation to improve the data's Gaussian distribution. Statistical analyses were conducted at the cluster level and utilized ANOVA to identify variations across the different groups. Only clusters that surpassed a cluster‐level threshold corrected at an FDR method of *p* < 0.05 were plotted.

### 2.10 Tissue Processing

At designated time points after injury, rats were anesthetized with isoflurane and were perfused transcardially with saline followed by 4% paraformaldehyde. Brains were removed, post‐fixed in paraformaldehyde for 48 h, routinely dehydrated, and embedded in paraffin. Brain samples embedded in paraffin were cut into coronal sections 5‐µm thick, ranging from −2.4 to −4.0 mm bregma, and subsequently utilized for Nissl staining and immunohistochemistry.

### 2.11 Nissl Staining

Paraffin‐sectioned tissue sections were dewaxed and dehydrated. Sections were stained with Nissl staining (ServiceBio Cat# G1036) for 2–5 min, followed by differentiation in 0.1% glacial acetic acid (Sinopharm Chemical Reagent, Cat# 10000218), and then washed with running water until colorless. Positive cells in the area with the maximal extent of brain regions with significant ReHo and FC alteration in fMRI results were counted by ImageJ software (https://imagej.net/ij/, RRID: SCR_003070) at a final magnification of 400×.

### 2.12 Immunohistochemistry

Paraffin‐embedded tissue sections were deparaffinized with xylene (Sinopharm Chemical Reagent, Cat# 10023418) and subsequently hydrated through a series of descending alcohol concentrations (100%, 90%, 80%, 70%, 50%, and pure water). For antigen retrieval, Tris‐ethylenediaminetetraacetic acid (EDTA) antigen repair buffer (ServiceBio Cat# G1203) was employed using the thermal repair method. Following this, sections were treated with appropriate amounts of endogenous peroxidase blocker (3% H_2_O_2_) and incubated for 25 min at room temperature (37°C). The sections were then washed three times with PBS buffer (3 min each) and incubated with 3% bovine serum albumin (BSA) (ServiceBio Cat# GC305010) for 30 min to inhibit nonspecific antibody binding. NeuN (1:500; ServiceBio Cat# GB11138, RRID: AB_2868432) and caspase‐3 (1:500; ServiceBio Cat# GB11532, RRID: AB_3096316) were the primary antibodies utilized in this study. Sections were incubated overnight at 4°C with primary antibodies, then treated with horse radish peroxidase‐labeled goat anti‐rabbit IgG (1:200; ServiceBio Cat# GB23303, RRID: AB_2811189) for 50 min. Chromogenic revelation was performed with a DAB kit (ServiceBio Cat# G1212). Counterstaining was conducted using hematoxylin. Positive cells in the area with the maximal extent of brain regions with significant ReHo and FC alteration in fMRI results were counted by ImageJ software (https://imagej.net/ij/, RRID: SCR_003070) at a final magnification of 200×.

### 2.13 Statistical Analysis

Statistical analysis was performed with IBM SPSS Statistics (version 26.0, IBM, RRID: SCR_002865). When the data met homogeneity of variance and normality, ANOVA was employed. When the assumptions of normality were not met, Kruskal–Wallis test was utilized. Correlation analysis adopted Spearman correlation analysis. A *p* < 0.05 was used to determine a significant distinction.

## 3 Results

### 3.1 ReHo Results

**FIGURE 1:**
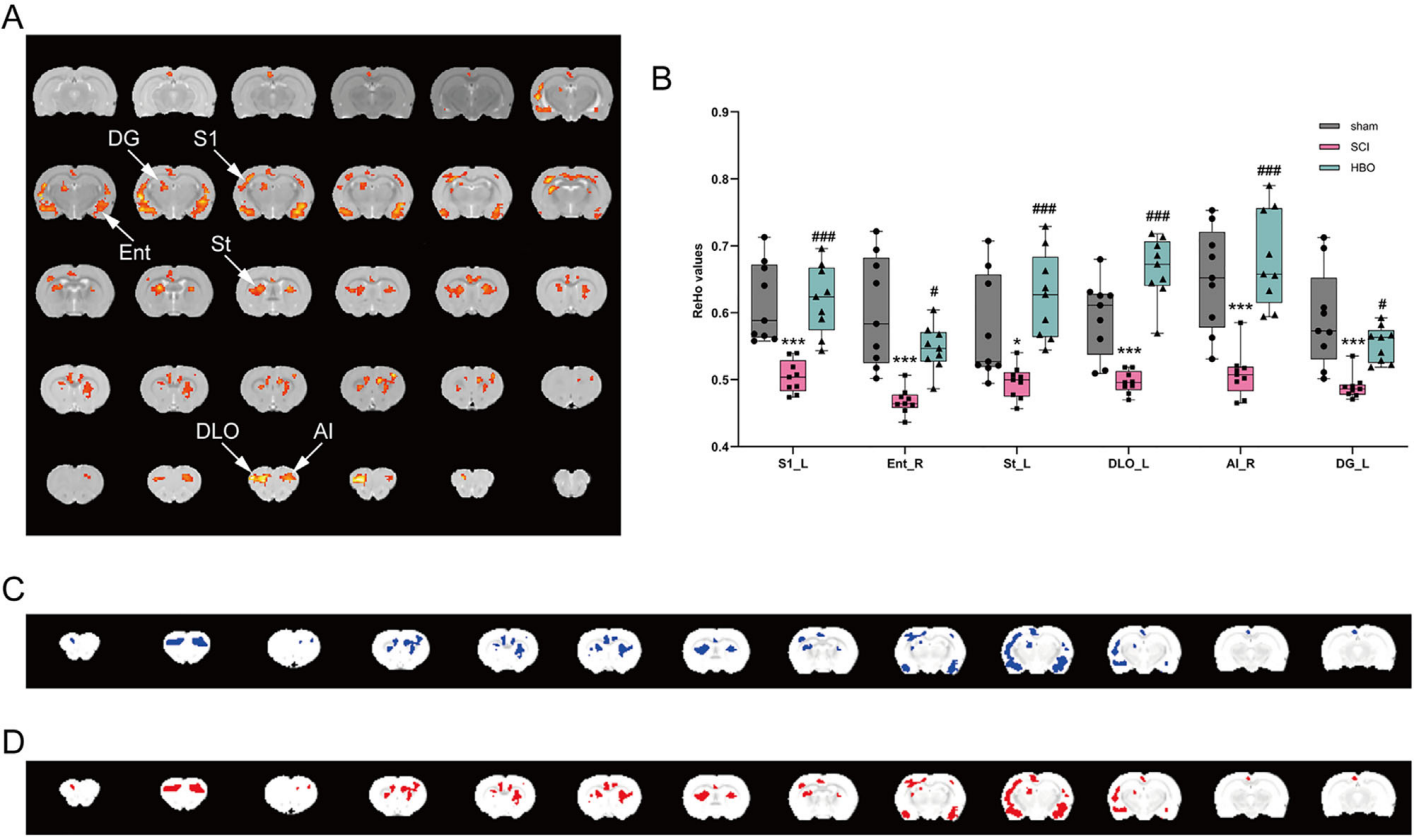
ReHo map shows HBO treatment promotes cerebral cortical activity in SCI rats. (A) Brain regions with significant ReHo alteration among the sham group, SCI group, and HBO group. Only clusters that surpassed a cluster‐level threshold corrected at a false discovery rate (FDR) method of *p* < 0.05 were plotted. (B) The statistical map depicts ANOVA differences in the ReHo values among the three groups. (C) The ReHo activities of the brain region decreased in the SCI group compared with the sham group. (D) The ReHo activities of the brain region increased in the HBO group compared with the SCI group. AI, agranular insular cortex; DG, dentate gyrus; DLO, dorsolateral orbital cortex; Ent, entorhinal cortex; L, left; R, right; S1, primary somatosensory cortex; St, striatum (*sham group vs. SCI group, *p < 0.05, **p < 0.01, ***p < 0.001. ^#^SCI group vs. HBO group, ^#^ *p* < 0.05, ^##^ *p* < 0.01, ^###^ *p* < 0.001).

Significant differences in ReHo values were observed among the sham group, SCI group, and HBO group, in the left primary somatosensory cortex (S1), right entorhinal cortex (Ent), left striatum (St), left dorsolateral orbital cortex (DLO), right agranular insular cortex (AI) and left dentate gyrus (DG). Post hoc comparisons were performed after extracting values from positive regions (showing significant differences). The SCI group exhibited significantly decreased ReHo values compared to the sham group. Conversely, the HBO group showed significantly increased ReHo values compared to the SCI group (*p* < 0.05, Figure 1).

### 3.2 FC Results

**FIGURE 2:**
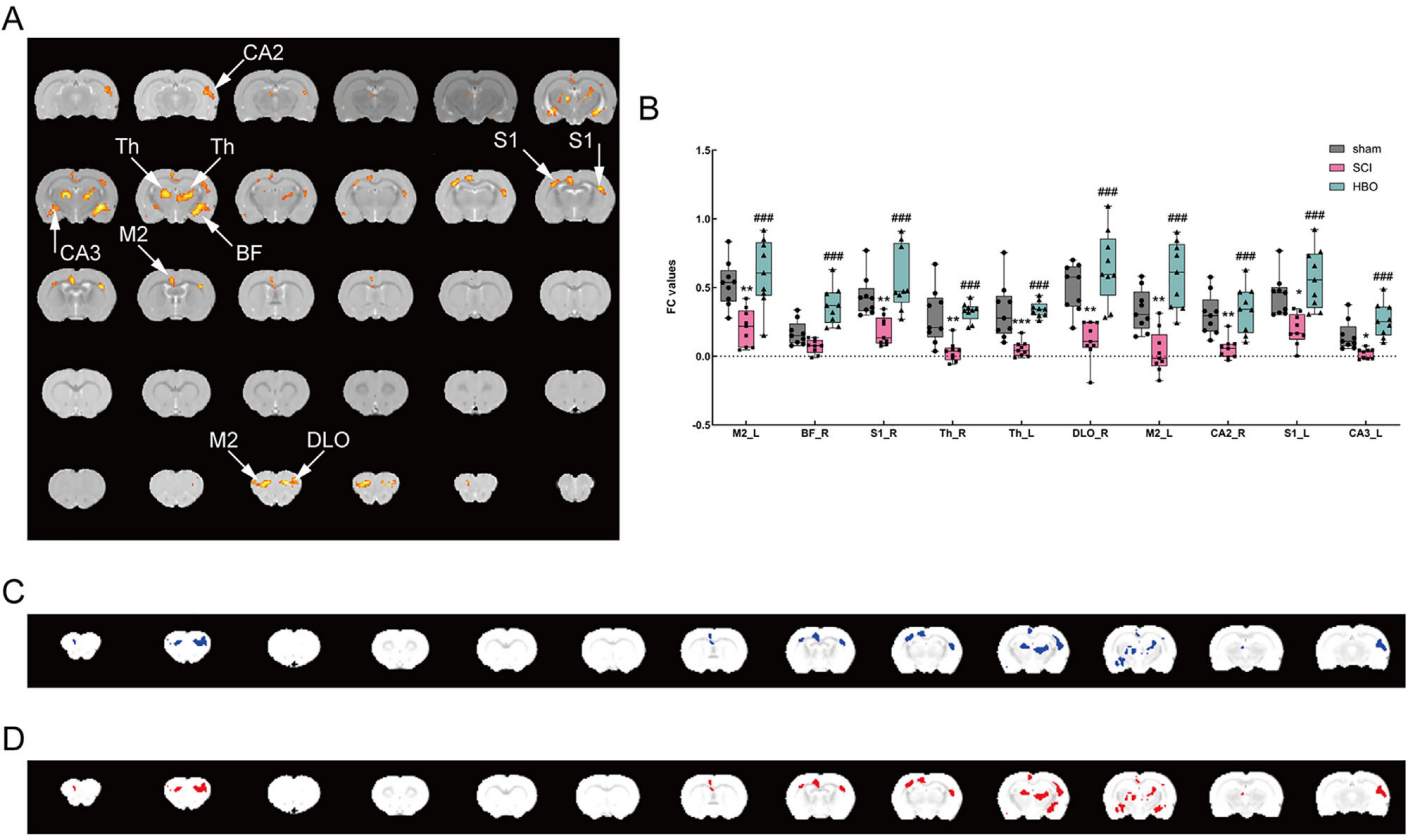
FC map shows HBO treatment increases functional connection strength between the motor cortex and certain brain regions in SCI rats. (A) Brain regions with significant FC alteration among the sham group, SCI group, and HBO group. Only clusters that surpassed a cluster‐level threshold corrected at an FDR method of *p* < 0.05 were plotted. (B) The statistical map depicts ANOVA differences in the FC strength among the three groups. (C) The FC strength of the brain region decreased in the SCI group compared with the sham group. (D) The FC strength of the brain region increased in the HBO group compared with the SCI group. BF, basal forebrain region; CA, cornu ammonis; DLO, dorsolateral orbital cortex; L, left; M2, secondary motor cortex; R, right; S1, primary somatosensory cortex; Th, thalamus (*sham group vs. SCI group, *p < 0.05, **p < 0.01, ***p < 0.001. ^#^SCI group vs. HBO group, ^#^ *p* < 0.05, ^##^ *p* < 0.01, ^###^ *p* < 0.001).

Voxel‐wised FC analysis was conducted with the motor cortex as the seed region. Significant differences in FC values were observed among the three groups in the left secondary motor cortex (M2), right basal forebrain region (BF), bilateral primary somatosensory cortex, bilateral thalamus (Th), right dorsolateral orbital cortex, right cornu ammonis 2 (CA2), and left cornu ammonis 3 (CA3). The cluster of peak MNI located in the left secondary motor cortex appeared twice. Post hoc comparisons were performed after extracting values from positive regions. SCI rats showed reduced FC values compared to the sham group, except for the right basal forebrain region, which showed no difference after SCI. Conversely, rising FC values were observed in all positive regions in the HBO group compared to the SCI group (*p* < 0.05; Figure 2).

### 3.3 Correlation Between Motor Performance and Neural Activity

**FIGURE 3:**
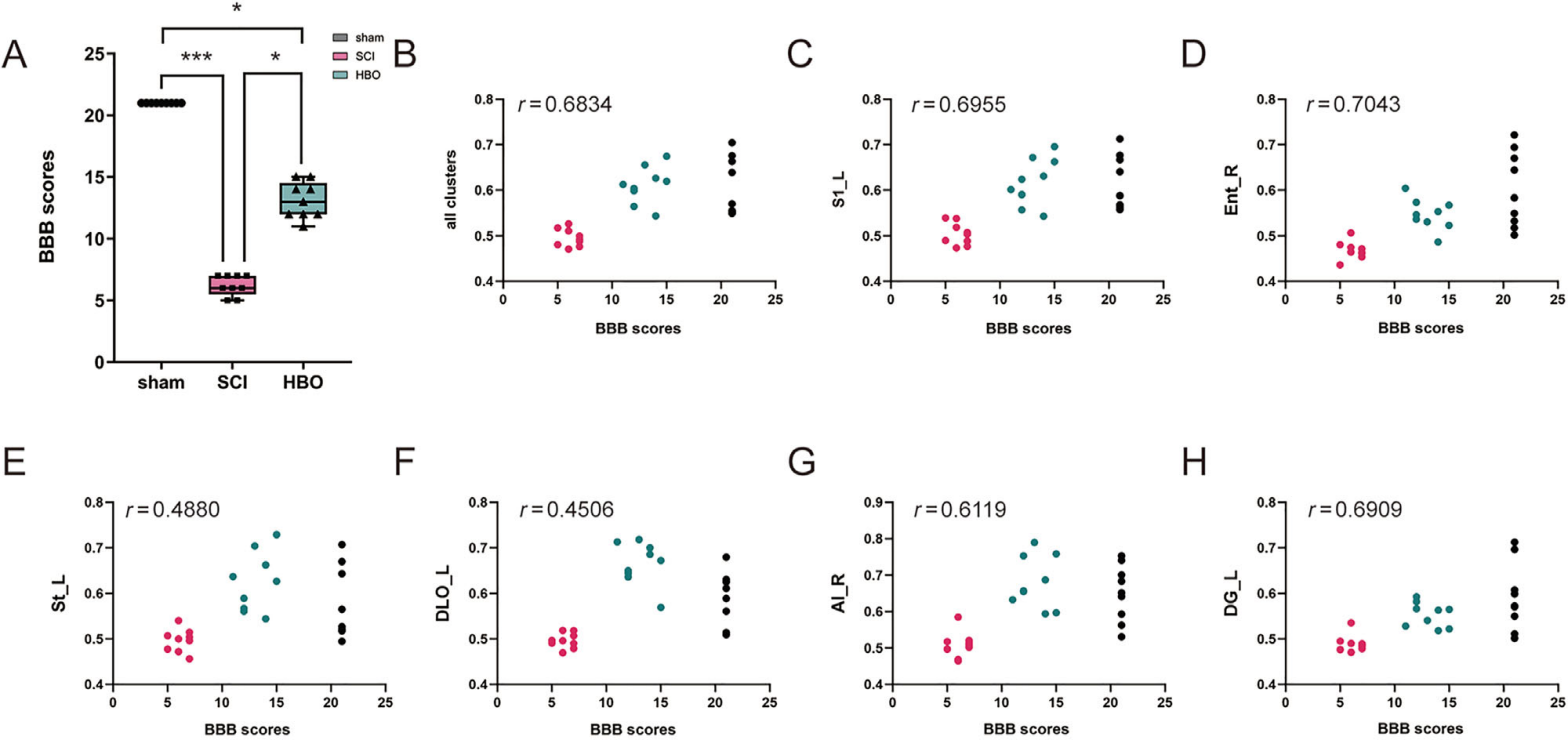
The ReHo values in all clusters were positively correlated with the BBB scores. (A) ANOVA differences in the BBB scores among the three groups (*p < 0.05, **p < 0.01, ***p < 0.001). (B–H) These images present the correlation between BBB scores and ReHo values in the respective brain region (*p* < 0.05). AI, agranular insular cortex; DG, dentate gyrus; DLO, dorsolateral orbital cortex; Ent, entorhinal cortex; L, left; R, right; S1, primary somatosensory cortex; St, striatum.

Motor function was assessed by measuring BBB scale scores, revealing reduced motor function in rats after SCI. HBO treatment showed potential in enhancing motor function recovery in SCI rats, but motor function scores remained significantly lower compared to those of the sham group (*p* < 0.05; Figure 3A).

Significant positive correlations were found between BBB scale scores and ReHo values in brain regions with significant ReHo alteration by Spearman correlation analysis in all clusters (*r* = 0.6834), left primary somatosensory cortex (*r* = 0.6955), right entorhinal cortex (*r* = 0.7043), left striatum (*r* = 0.4880), left dorsolateral orbital cortex (*r* = 0.4506), right agranular insular cortex (*r* = 0.6119), and left dentate gyrus (*r *= 0.6909), respectively (*p < *0.05; Figure 3B–H).

**FIGURE 4:**
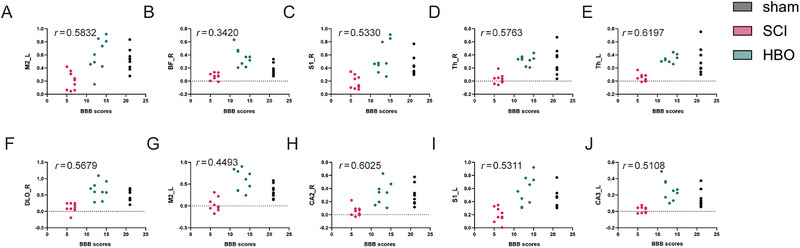
The FC values in all clusters were positively correlated with the BBB scores. (A–H) These images present the correlation between BBB scores and FC values in the respective brain region (*p* < 0.05). BF, basal forebrain region; CA, cornu ammonis; DLO, dorsolateral orbital cortex; L, left; M2, secondary motor cortex; R, right; S1, primary somatosensory cortex; Th, thalamus.

The strengths of abnormal FC mentioned above were extracted from each rat of these three groups, and the correlation of the FC strength versus BBB scale score was individually analyzed. Spearman correlation analysis released positive correlations between motor function and the FC strength between the motor cortex and other regions, including the left secondary motor cortex (*r*1 = 0.5832, *r*2 = 0.4493), right basal forebrain region (*r* = 0.3420), right primary somatosensory cortex (*r* = 0.5330), right thalamus (*r* = 0.5763), left thalamus (*r* = 0.6197), right dorsolateral orbital cortex (*r* = 0.5679), right cornu ammonis 2 (*r* = 0.6025), left primary somatosensory cortex (*r* = 0.5311), and left cornu ammonis 3 (*r* = 0.5108) (*p* < 0.05; Figure 4).

### 3.4 Pathological Changes

**FIGURE 5:**
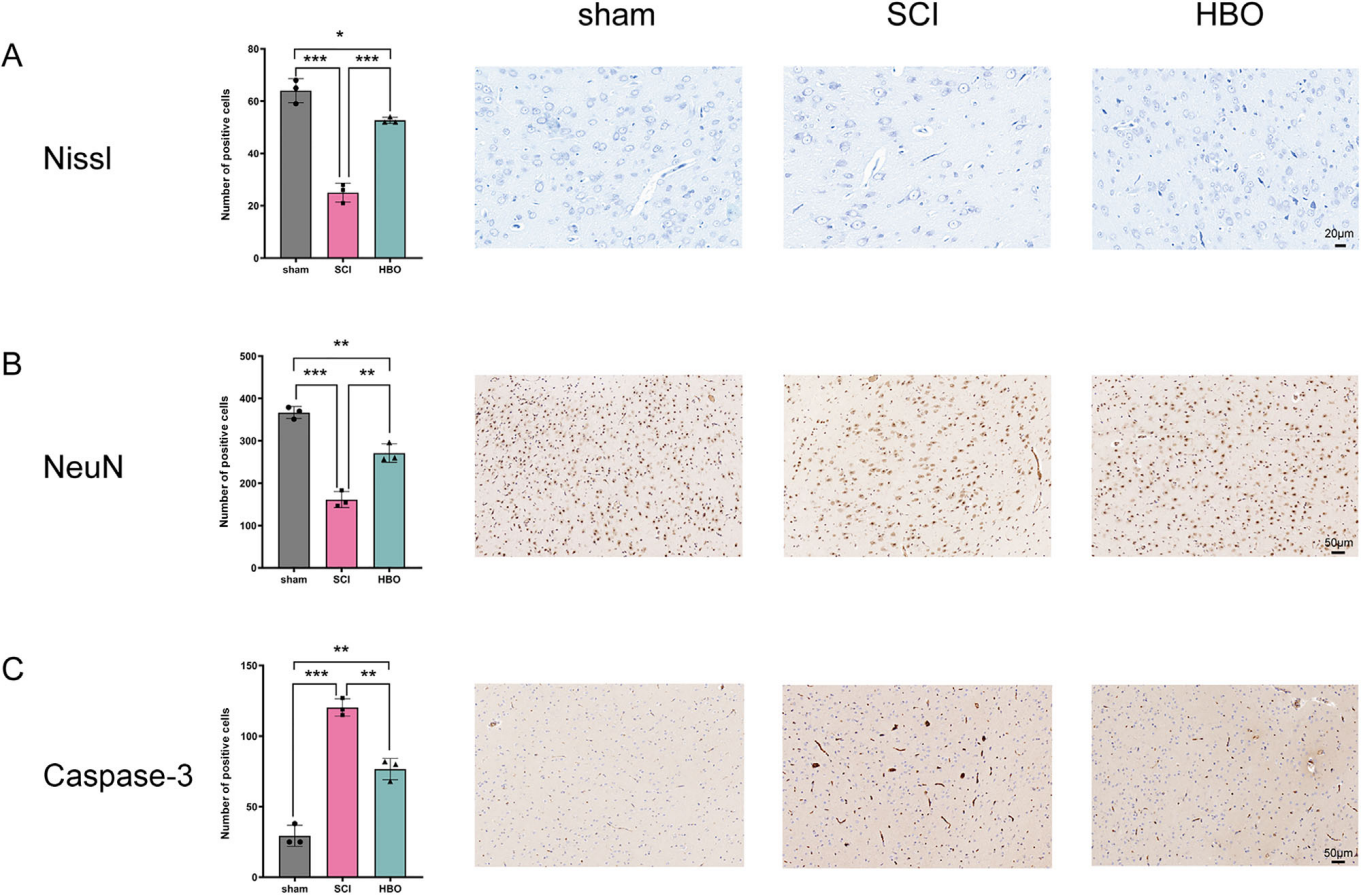
Histopathological changes in brain regions with significant ReHo and FC alteration in different groups (*p < 0.05, **p < 0.01, ***p < 0.001).

Our study found a significant reduction in the number of Nissl body and NeuN positive cells in SCI group rats compared to those in the sham and HBO groups (*p* < 0.05; Figure 5A,B). In addition, the number of caspase‐3 positive cells was significantly higher in rats with SCI than those in the sham and HBO groups (*p* < 0.05; Figure 5C).

## 4 Discussion

### 4.1 HBO Treatment Protocol Selection for SCI Rats

In clinical treatments of HBO for SCI, 2.0 ATA is a commonly selected treatment pressure, and many animal studies have also chosen this plan (Z. C. Sun et al. 2022; Z. Zhang et al. 2022; Ying et al. 2019; Feng and Li 2017). Therefore, we selected a relatively standard treatment pressure for our preliminary study. Furthermore, aside from conservative HBO treatment (once a day), an intensified regimen of therapy (several times a day) is another treatment approach. The intensified therapy can enhance the oxygen environment in SCI rats through increased treatment frequency, thereby reducing inflammation, inhibiting apoptosis, and promoting recovery of motor function. However, in clinical practice, multiple treatments may lead to increased costs, poor patient compliance, and side effects such as seizures or pulmonary oxygen toxicity. These considerations guided our choice of a conservative HBO treatment protocol in the current study.

### 4.2 HBO Treatment Promotes Cerebral Cortical Activity in SCI Rats

Previous studies have shown widespread brain functional abnormalities following SCI. Some research has indicated SCI could reduce neuron activity in the motor cortex and somatosensory cortex, whereas others reported similar or increased activation levels could be seen (Zhu et al. 2015; Rao et al. 2015; Q. Chen et al. 2018). Discrepancies in these findings may result from variations in injury severity, levels, time, and treatment methods among clinical SCI patients. Consequently, this study aims to mitigate bias among subjects in previous studies by establishing a reproducible SCI rat model.

Rao et al. (2013) observed decreased functional activation in primary somatosensory cortex during the chronic phase of SCI, with no subsequent functional recovery over time. Our findings support and extend previous research, confirming lower primary somatosensory cortex activation in the early stage of SCI. Moreover, the strength of neuronal activation in primary somatosensory cortex in SCI rats with HBO treatment was higher than that in untreated SCI rats, indicating that HBO treatment can promote the recovery of primary somatosensory cortex function.

The limbic lobe comprises the hippocampus, parahippocampal gyrus, dentate gyrus, cingulate gyrus, and part of cortical regions related to olfactory function (Sitoh and Tien 1997). These structures, along with analogous regions in the limbic lobe and certain subcortical structures sharing close functional connections, collectively form the limbic system. The limbic system is generally considered to be involved in visceral activity, emotional memory, and other functions and can connect behavior and cognitive functions (Catani, Dell'acqua, and Thiebaut de Schotten 2013; Rolls 2019). In this study, significant alterations were observed in the cortical functional activation of limbic system structures, including the right entorhinal cortex, left dentate gyrus, and left dorsolateral orbital cortex, showed significant changes after SCI and HBO treatment. Positioned in the medial temporal lobe, the hippocampus plays a crucial role in spatial navigation, memory formation, and learning behavior. Previous research has shown that the transmission of information from the cerebral cortex to the hippocampus occurs via the entorhinal cortex–dentate gyrus–cornu ammonis 3–cornu ammonis 1 pathway, and the relevant neuron activity in this pathway is the basis for learning behavior (Buzsáki and Moser 2013; Li et al. 2017).

The dorsolateral orbital cortex is located in the orbitofrontal cortex and belongs to the limbic system. It plays a critical role in visuospatial processing, as well as in reward processing and reward‐based learning (Izquierdo 2017). Combined with our results, we hypothesize that the loss of afferent sensory and efferent motor pathways after SCI may cause the decline of spatial navigation and learning abilities in rats, whereas HBO treatment may partially restore limbic system functions to alleviate reduced motor function.

The striatum is the main input structure of the basal ganglia and is involved in motor control and executive functions, such as motor learning, behavioral control, and emotion (Janssen et al. 2015). The study by M. Suzuki et al. (2020) on a macaque SCI model found that striatal function can decline after SCI and is a key node of the cortical reorganization required for functional recovery of fine movement skills. Similarly, our study demonstrated a decline in striatal function, with subsequent enhancement in striatal activation strength following HBO treatment concurrent with motor function recovery.

In addition, we observed activation of the decreased insular cortex after SCI, consistent with Q. Chen et al. (2017)'s findings. Agranular insular cortex is located within the insular cortex, along with the anterior cingulate cortex, forming a “salience network” that guides behavior by segregating the most relevant among internal and extrapersonal stimuli (Menon and Uddin 2010). Consequently, functional changes in agranular insular cortex may indicate alterations in related brain areas resulting from functional loss after SCI, with some cortical functions partially restored following HBO treatment, thereby facilitating motor function recovery.

### 4.3 HBO Treatment Increases Functional Connection Strength Between the Motor Cortex and Whole‐Brain Voxels in SCI Rats

Using the motor cortex as a seed, voxel‐wised FC analysis assessed alterations in whole‐brain FC of brain regions. The SCI group showed decreased FC strength in the left secondary motor cortex, bilateral primary somatosensory cortex, bilateral thalamus, right dorsolateral orbital cortex, right cornu ammonis 2, and left cornu ammonis 3 compared with the sham group, whereas the HBO group demonstrated increased FC strength in the aforementioned brain regions, as well as in the right basal forebrain region, compared with the SCI group.

The basal forebrain region comprises brain structures located in the rostroventral forebrain, traditionally defined by the presence of cholinergic projection neurons crucial for regulating arousal, attention, learning, and memory processes (Hangya et al. 2015; Wang et al. 2021). Our study revealed insignificant functional connection strength between the right basal forebrain region and the motor cortex changes after SCI, whereas enhancement of FC strength and improvement of motor function could be seen after HBO treatment. These results indicate that the learning function of the basal forebrain region may remain unaffected by motor function loss due to apraxia. However, after HBO treatment, they also suggest that SCI rats could potentially regain motor function through compensatory FC in the basal forebrain region‐motor cortex.

Motor and somatosensory functions can be lost below the level of injury after SCI. Furthermore, our research also demonstrated the decreased strength of FC between the motor cortex and primary somatosensory cortex with the reduction of motor function. By the cerebral cortex–basal ganglia–cerebral cortex circuit, information from the somatosensory cortex and motor cortex is transmitted to the basal ganglia and then fed back to the primary somatosensory cortex and secondary motor cortex of the cerebral cortex via thalamus, jointly completing sensorimotor transformations (Oury et al. 2006; Svoboda and Li 2018; Yang and Kwan 2021; J. Liu et al. 2023). Diminished sensory input may diminish somatosensory cortex function, which leads to reduced functional connectivity (FC) of the thalamus and secondary motor cortex through this loop. In addition, partial motor function restoration observed in HBO‐treated SCI rats is accompanied by enhanced FC strength of the motor cortex to the bilateral thalamus, bilateral primary somatosensory cortex, and left secondary motor cortex. These findings imply a crucial role for the cerebral cortex–basal ganglia–cerebral cortex circuit in affecting motor function.

Previous research has shown that the functions of cornu ammonis 2 and cornu ammonis 3 are associated with encoding new information (Suthana et al. 2011). Therefore, when motor function declines, the strength of FC between the motor cortex and cornu ammonis 2 can also decrease due to the loss of new information to process. HBO treatment not only promotes the recovery of motor function but also enhances the motor cortex's connectivity to cornu ammonis 2 and cornu ammonis 3. This finding suggests that, during motor function recovery, increased FC strength in brain regions related to new information processing may compensate for the need to learn new movement patterns and enhance limb control. In addition, the function of the dorsolateral orbital cortex is associated with learning abilities and can influence behavior through projections to secondary motor cortex (Gerbella et al. 2010; Saleem, Miller, and Price 2014; Izquierdo 2017; Rolls et al. 2020). Therefore, the loss of previous motor functions and the acquisition of new motor functions will also lead to alterations in the strength of FC between the motor cortex and dorsolateral orbital cortex.

### 4.4 ReHo and FC Are Reliable Neuroimaging Indexes to Reflect Motor Functional Changes

This study investigated changes in regional brain activities and FC at resting state during the early stage of SCI rats and their correlation with motor function alterations. Rat hind limb movement was assessed using the BBB scale scores. The findings revealed a near‐complete loss of hind limb movement in SCI rats compared to sham animals, with HBO treatment facilitating hind limb motor function recovery in SCI rats. ReHo and FC values in all clusters were positively correlated with the BBB scale scores, suggesting a close association between local brain activity changes and FC with the motor ability of rats. It indicates that the higher ReHo and FC values correspond to the higher motor function.

### 4.5 HBO Treatment Reduces Apoptotic Proteins, Increases the Number of Neurons, and Protects Neuronal Function in Brain Regions With Significant ReHo and FC Alteration in SCI Rats

To further investigate the mechanism by which HBO treatment promotes the degree of brain activation and recovery of motor function in SCI rats, Nissl staining and immunohistochemical staining of NeuN and caspase‐3 were performed in brain regions with significant ReHo and FC alteration. The status of neurons can be indicated by Nissl body as they can shrink or disappear when neurons are over‐stimulated (Kakinohana et al. 2011; Kanemaru et al. 2023). The anti‐NeuN antibody specifically recognizes the nucleus and cytoplasm of most neurons, so it can be used to assess the number of neurons (Q. Zhang et al. 2023). Caspase‐3 is a key molecule in apoptosis, and its formation marks irreversible apoptosis (Yin et al. 2019; Romero‐Ramírez et al. 2020). Our study showed a decrease in Nissl body and NeuN positive cells after SCI, which were seen to increase after treatment with HBO. In contrast, caspase‐3 positive cells were significantly increased in the SCI group, and their expression decreased after HBO treatment. This result suggests that after SCI, not only the brain function but also the brain pathological structure changes, manifested by the decrease of neuron number, the decrease of neuron function, and a large number of cells into programmed death. In contrast, HBO treatment reduced neuronal damage and apoptosis. This result also showed good consistency with our fMRI results, confirming that changes in neuronal number and function can cause changes in cortical activity. It reveals that HBO treatment may promote the recovery of motor function by inhibiting apoptosis in the cerebral cortex.

Our study had several limitations worth noting. First, we referenced previous studies to determine our sample size, which we believe meets the preliminary experimental requirements (Xiao et al. 2023; Sanganahalli et al. 2021, 2022, 2021, 2022). However, compared to other experiments, there is still the issue of a relatively small sample size. We plan to further increase the sample size in future studies when conditions permit to enhance the statistical power of our experiments. Second, based on previous studies, we chose a conservative HBO treatment protocol of 2.0 ATA with daily treatment (Siglioccolo et al. 2024). However, we did not investigate the effects of different pressure gradients and more frequent treatments on HBO efficacy. Future studies can add more pressure gradients and an enhanced treatment plan with increased treatment frequency. Third, integrating advanced neuroimaging techniques and molecular assays could be further refined in the future.

## 5 Conclusion

In conclusion, this study reveals that HBO treatment facilitates both functional and structural plasticity in the brain, contributing to the recovery of motor function in rats with SCI.

## Author Contributions

**Xinyi Yang**: methodology, writing–original draft. **Zhongyue Wu**: formal analysis. **Huimin Lai**: formal analysis. **Lingling Chen**: formal analysis. **Dairong Cao**: methodology, funding acquisition, writing–review and editing. **Fang Liu**: methodology, funding acquisition, writing–review and editing.

## Ethics Statement

The authors affirm that this study adheres to the ethical standards for Brain and Behavior and have received approval from the Ethics Committee of the Fujian Medical University (IACUC FJMU 2022‐0879).

## Conflicts of Interest

The authors declare no conflicts of interest.

### Peer Review

The peer review history for this article is available at https://publons.com/publon/10.1002/brb3.70196.

## Data Availability

The data that support the findings of this study are available from the corresponding author upon reasonable request.
